# The use of choline supplementation in premature newborns: a systematic review

**DOI:** 10.1590/1984-0462/2025/43/2024214

**Published:** 2025-08-08

**Authors:** Ligia Modelli Rodrigues, Allan Chiaratti de Oliveira, Tulio Konstantyner

**Affiliations:** a Universidade Federal de São Paulo, São Paulo, SP, Brazil.

**Keywords:** Choline, Premature infant, Neurodevelopmental disorders, Parenteral nutrition solutions, Enteral nutrition, Colina, Recém-nascido prematuro, Transtornos do neurodesenvolvimento, Soluções de nutrição parenteral, Nutrição enteral

## Abstract

**Objective:**

To identify the routes and doses of choline supplementation required to meet the metabolic demands of preterm infants.

**Data Source:**

The information was searched in three databases: US National Library of Medicine National Institute of Health (PubMed), Scientific Electronic Library Online (SciELO) and Web of Science. The search for articles was updated in August 2024, with no restrictions on publication year or language. This review was registered in the International Prospective Register of Systematic Reviews — PROSPERO (ID: 549568).

**Data synthesis:**

The final selection of studies yielded eight original articles, which were subsequently evaluated for their methodological quality. The recommended dosage of parenteral choline remains uncertain. However, studies have demonstrated a reduction in serum choline level inversely proportional to the quantity administered via parenteral nutrition, particularly in preterm infants with slow progression to enteral nutrition. The dose of 40–50 mg/kg/day of enteral choline appears to be sufficient to maintain plasmatic levels at a concentration comparable to that observed in the umbilical cord of preterm infants.

**Conclusions:**

The need for daily choline supplementation from the first day of life is still controversial. The results of the selected studies do not allow for the determination of the optimal choline dosage for parenteral nutrition in these infants. Nevertheless, there is some scientific evidence suggesting that providing 40–50 mg/kg/day enterally is sufficient to meet the metabolic needs of preterm infants.

## RESUMO

**Objetivo:**

Identificar as vias e doses de suplementação de colina necessárias para a demanda metabólica de recém-nascidos prematuros.

**Fontes de dados:**

A busca das informações foi realizada em três bases de dados: US National Library of Medicine National Institute of Health (PubMed), Scientific Electronic Library Online (SciELO) e Web of Science. A busca por artigos foi atualizada em agosto de 2024, sem restrições de ano ou idioma. Esta revisão foi registrada pelo *International Prospective Register of Systematic Reviews* — PROSPERO (ID: 549568).

**Síntese dos dados:**

A seleção final dos estudos resultou em oito artigos originais, que foram avaliados quanto à sua qualidade metodológica. A dose recomendada de colina por via parenteral ainda é incerta, mas estudos mostraram a diminuição da concentração sérica de colina de forma inversamente proporcional à quantidade fornecida pela nutrição parenteral, principalmente nos prematuros com avanço lento da nutrição enteral. A dose de 40 a 50 mg/kg/dia de colina via enteral parece ser capaz de manter os níveis plasmáticos próximos à quantidade de colina presente no cordão umbilical do prematuro.

**Conclusões:**

A necessidade de suprimento diário de colina desde o primeiro dia de vida ainda é controversa. Os resultados dos estudos selecionados não permitem concluir qual a quantidade de colina que deve ser oferecida na nutrição parenteral. No entanto, há algumas evidências científicas sugestivas de que fornecer 40–50 mg/kg/dia por via enteral é suficiente para atender às necessidades metabólicas destes recém-nascidos.

## INTRODUCTION

 Choline (N-trimethylethanolamine) is a quaternary amine that is synthesized endogenously from the amino acid methionine and is a substrate for the synthesis of phosphatidylcholines, which constitute 40 to 50% of cell membranes and 70 to 95% of the phospholipids present in surfactant, bile and lipoproteins.^1-3^ This essential nutrient in the human body is a precursor of acetylcholine, which plays a role in the formation of neurons, synapses, and dendritic trees during the fetal and neonatal period. It also promotes the proliferation and apoptosis of stem cells and participates in the transport and metabolism of lipids and cholesterol.^1^ In addition, choline can be metabolized to betaine, an alternative methyl donor in homocysteine remethylation, participating in the regulation of homocysteine-methionine metabolism and in the methylation of macromolecules, such as DNA and histones, possibly impacting epigenetic signature.^2-4^

 Fetal/perinatal choline deficiency has a deleterious impact on the neurogenesis of newborns, with long-term consequences including memory disorders and impaired function of the liver, kidneys, and pancreas.^1^ In this context, experimental studies employing pre- and post-natal choline supplementation in rodents, healthy and in disease models, such as fetal alcohol syndrome, Rett syndrome, and Down syndrome, have yielded evidence supporting the benefits of choline supplementation on hippocampal development and subsequent improvements in learning behavior and memory.^5^

 Although choline is an essential dietary nutrient with recommended intake levels, its dietary requirement remains controversial.^2 ,5-7^ The US National Academy of Sciences recommends a daily enteral intake of 18 mg/kg or 125 mg for infants aged zero to six months. However, there is no specific recommendation for preterm infants, who need higher amounts due to the exceptionally high growth rate of this risk group.^1,8^ Although choline can be endogenously synthesized, this process would normally not be enough to meet the metabolic demand of preterm infants. Therefore, dietary intake or supplementation would be necessary.^2,8^

 The supposition that current feeding regimens are inadequate for preterm infants regarding choline supply is corroborated by studies indicating that the estimated adequate intake of choline for very and extremely low birth weight infants is not attained.^1^ Furthermore, plasma concentrations of choline decline precipitously following preterm birth, due to the cessation of this nutrient’s supply via the umbilical cord.^9^

 The number of surviving children born prematurely has increased substantially over the last two decades, which has created challenges in the clinical and nutritional management of these newborns. Despite evidence supporting the need for choline supplementation in preterm infants and the potential neurodevelopmental benefits of supplementing this nutrient at appropriate doses and times, the optimal dosage, timing, and administration of choline for these newborns remain unclear.^10^

 In this context, this study aimed to identify the routes and doses of choline supplementation needed to meet the metabolic demands of premature newborns. 

## METHOD

 This study is a systematic review of scientific articles selected in accordance with the Preferred Reporting Items for Systematic Reviews and Meta-Analyses (PRISMA) guidelines. The bibliographic search was conducted in the following databases: US National Library of Medicine, National Institutes of Health (PubMed), Scientific Electronic Library Online (SciELO), and Web of Science, with no limitations on year or language. Articles published up to August 13, 2024, were selected. This review has been registered with the International Prospective Register of Systematic Reviews (PROSPERO ID: 549568). 

 To define the research question that guided the search for pertinent articles and defined the inclusion criteria, the "patient, intervention, comparison, outcome" (PICOS) strategy was employed: (P) preterm infants of any gestational age were defined as participants; (I) the intervention studied was of choline supplementation, received in any amount and time of administration; (C) groups of newborns with no choline supplementation; (O) the outcomes chosen were serum choline or phosphatidylcholine concentrations any time after supplementation; and (S) original studies with no limitations on design characteristics (experimental or observational). 

 The search for articles used a combination of the Medical Subject Headings (MeSH) terms: "choline" and "infant, premature", which initially resulted in 118 and 47 articles in the PubMed and Web of Science databases, respectively. A search of SciELO yielded no results when the two selected descriptors were entered. Next, 13 duplicate articles between the databases were excluded, leaving 152 articles. Of these, articles that did not contain sufficient information to address the research question, review articles, letters to the editor, editorials, book chapters, series or case reports, and animal studies were excluded. 

 This stage was conducted by two examiners, who independently decided to exclude the studies because they were not original, did not provide information related to the research question or did not meet the methodological requirements described by the respective evaluation scales. Disagreements between the examiners were discussed and resolved together. 

**Figure 1:**
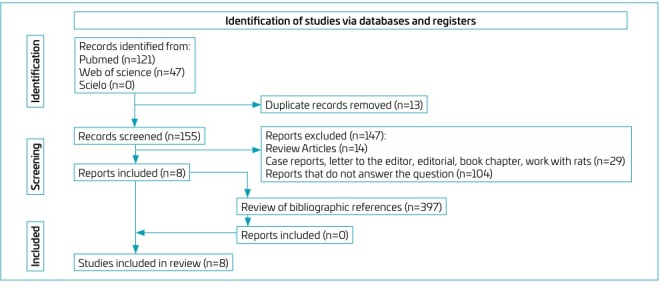
Flowchart of the article selection process.

 Thus, eight articles were selected. As an additional strategy for identifying relevant studies, the 397 references cited in the articles selected during the inclusion stage were analyzed for methodology and content using the same criteria employed in the selection and eligibility stages. Figure 1 illustrates the process of selecting scientific articles. 

 The eight selected studies underwent a methodological quality check using the Newcastle-Ottawa Scale (NOS). This scale was used for the seven cohort studies, which included three dimensions of analysis (selection, comparability, and exposure/outcome) and assessment of the risk of bias according to the Cochrane Collaboration criteria for the only clinical trial included, in which the seven domains are analyzed: random sequence generation, allocation concealment, blinding of participants and the professional, blinding of outcome assessors, incomplete results, selective outcome reporting and other sources of bias. 

 Meta-analysis was not possible in this review because of the heterogeneity of the selected studies (methods, study populations, interventions, outcomes and follow-up periods) and the different types of outcomes assessed in these studies. 

## RESULTS

 The selection process resulted in eight articles which met the established criteria and answered the question posed in this study.^1 ,7,9,11-15^ These articles included seven cohort studies and one randomized clinical trial. Notably, 50% (n=4) of the studies were conducted by the same group of researchers from EberhardKarls-University in Germany. 

 All cohort studies were classified as having "good" methodological quality in the three dimensions of analysis of the Newcastle-Ottawa scale (selection, comparability, and exposure/outcome), as presented in Table 1. The single clinical trial had a "low" risk of bias in all the seven domains assessed according to the criteria of the Cochrane Collaboration. 

**Table 1:** Results of the methodological quality analysis for each of the seven cohort studies included in the systematic review (NOS tool).

Studies	Items								
	Selection				Comparability	Exposure			Score
	1	2	3	4	1	1	2	3	
Nilsson et al.^11^	-	*	*	*	**	*	*	*	8/9
Goss et al.^12^	-	*	*	*	*	*	*	*	7/9
Maas et al.^13^	-	-	*	*	**	*	*	*	7/9
Bernhard et al.^1^	-	*	*	*	**	*	*	*	8/9
Bernhard et al.^14^	-	*	*	*	**	*	*	*	8/9
Holmes et al.^7^	-	*	*	*	**	*	-	*	7/9
Alexandre-Gouabau et al.^15^	-	*	*	*	**	*	*	*	8/9

NOS: Newcastle-Ottawa Scale.

Selection: 1*somewhat representative of the average in the community; 2*drawn from the same community as the exposed cohort; 3*secure record (eg surgical records); 4* demonstration that outcome of interest was not present at start of study.

Comparability: 1*study controls for (select the most important factor), 1**study controls for any additional factor (this criterion could be modified to indicate specific control for a second important factor).

Exposure: 1* independent blind assessment; 2* was follow-up long enough for outcomes to occur; 3* complete follow up-all subjects accounted for or subjects lost to follow up unlikely to introduce bias-small number lost.

 Most studies showed outcomes that support the importance of maintaining optimal choline levels and the need for choline supplementation in premature newborns. 

 The clinical trial was conducted with the objective of assessing the effects of choline supplementation in 24 neonates born at a gestational age between 24 and 31 weeks and aged between eight and ten days. The trial recommended administering choline chloride via the enteral route at a dose of 30 mg/kg/day. This regimen resulted in a combined enteral supply (supplementation + diet) of 35–85 mg/kg/day, which was necessary to achieve a plasma choline concentration comparable to that observed in the umbilical cord (41.4 μmol/L).^9,14^

 The provision of choline via parenteral nutrition in premature infants has also been evaluated, demonstrating the consequent risk of choline deficiency. This is because the parenteral formulation used in the studies provided no or insufficient amount of choline in their composition.^11^ Predominant parenteral nutrition led to a progressive decrease in plasma choline concentrations in the first seven days of life.^11^ Similarly, Bernhard et al. investigated the effects of enteral diets with choline supplementation in conjunction with parenteral nutrition devoid of choline. Their findings revealed that only 1 to 2% of infants attained an optimal choline intake due to the limited amount provided, a consequence of the immature digestive system.^1^

 Other studies have evaluated the concentration of choline in breast milk and have shown variations over the course of days of life. In the study by Maas et al., the median choline content of breast milk from mothers of premature infants (158 mg/L) was observed to be lower in comparison to milk from mothers of full-term infants (258 mg/L).^13^ Another study demonstrated a higher concentration of choline in mature breast milk compared to colostrum, which supports the notion that the demand for choline increases with developmental progression.^7^ The levels of choline in the plasma of premature infants under 28 weeks of gestational age were compared to those in the umbilical cord. The findings revealed that the plasma choline levels declined to approximately 50% of the umbilical cord concentrations within 48 hours of birth, from 41.4 to 20.8 μmol/L. The results of this study demonstrated a correlation between the degree of prematurity and the likelihood of developing choline deficiency in infants.^14^

 Another cohort study demonstrated that the endogenous phosphatidylcholine production pathway (catalyzed by phosphatidylethanolamine-N-methyltransferase, or PEMT) is inadequate to maintain its plasma concentration.^11^ The increase in phosphatidylcholine (of which choline is a precursor) in the first five days of life was promoted by intravenous supplementation of 3.6 mg/kg of deuterated methyl-D9-choline chloride 48 hours after birth. Isotopic enrichment studies demonstrated that phosphatidylcholine was predominantly produced by direct choline incorporation via the cytidine diphosphate-choline (CDP-choline) pathway.^12^

 The study by Alexandre-Gouabau et al. evaluated the impact of choline concentration in breast milk on weight gain in preterm infants born between 27 and 34 weeks who received only breast milk as an enteral diet for a period of 28 days. It was observed that infants who received milk with a higher concentration of choline exhibited accelerated and more optimal growth during the first month of life (median of 2.67 x 10^6^ ions des choline in the slow growth group versus a median of 3.30 x 10^6^ ions des choline in the fast growth group).^15^

 The methodological characteristics and results of interest of the eight selected studies are shown in Table 2. 

**Table 2:** Methodological characteristics of choline supplementation in premature infants, according to the selected studies.

Studies	Design and population	Method	Results
Holmes et al.^7^ , England	Cohort8 NB between 28μ38 weeks, in BM	Amount of choline in colostrum (2 to 6 days), BM (7 and 22 days) (Proton NMR spectroscopy)	Mean choline: colostrum (0.10 mmol/L) versus BM (0.21 mmol/L): p<0.05
Bernhard et al.^1^ , Germany	Cohort (MP: 98 days)62 NB <1000g or <28 weeks, in PN + EN	Estimated choline requirement adjusted for weight Choline content in the BM, IF and PN offered	↑ choline requirement as ↓ birth weight (Md: 27.4 mg/kg/day — weight 735 g)Diet met intake requirements on day 11 in 50% of NB
Bernhard et al.^14^ , Germany	Cohort (MP: 84 days) 176 NB (24–42 weeks)	162 blood samples from 56 PTNB <1500 g176 cord blood samples Plasma choline (tandem MS)	[choline] PTNB: UC (41.4 μmol/L) versus 48h DL (20.8 μmol/L): p<0.001
Maas et al.^13^ , Germany	Cohort (MP: 4 months)353 BM samples from PTNB mothers X 9 FNB mothers	Plasma choline in BM (tandem MS and CG) — collected in the first 4 months	Md choline: mothers PTNB (158 mg/L) versus FNB (258 mg/L): p<0.001
Alexandre-Gouabau et al.^15^ , France	Cohort NB between 27–34 weeks PN + BM for 28 days	Groups separated by weight gain between birth and discharge38 NB SGG versus 29 NB FGGBM: HPLC-MS	Md Choline ions: SGG (2.67x10^6^) versus FGG (3.30x10^6^): p=0.008
Bernhard et al.^9^ , Germany	RCT (MP: 10 days)24 RN (GA 24–31 weeks) 8 DL, on EN	IG: standard diet + choline chloride (30 mg/kg/d) for 10 days versus CG: standard diet7th day: 3.6 mg/kg enteral D9-choline chloride (isotope marker) Plasma [choline] before intervention and at 8–10 DL (12h + 60h after D9-choline dose)	M_d_ [choline] before intervention: IG versus CG: p>0.05↑ M_d_ [choline] (μmol/L) on D8–10: IG (35.4) versus CG (17.8): p<0,05
Nilsson et al.^11^ , Sweden	Cohort (MP: 28 days)87 NB <28 weeks, on PN + EN	Plasma [choline] (MS): 1, 7, 14 e 28 DL Start PN or minimum EN: between 6 and 12h DL	M_d_ [choline] (μM): D1 (33.7) versus D7 (18.4): p<0.05 M_d_ [choline] (μM): After day 7: slow increase, but <day 1
Goss et al.^12^ , England	Cohort (MP: 10 days)31 NB (GA <28 weeks), <48h DL, <1250 g, in OIT and PN (1.04 mg/ml of choline)	1st dose 3.6 mg/kg D9-choline chloride IV in the first 48h DL2nd dose (equal) in intubated patients after 120h DL (n=10) Plasma [PC]: before IV supplementation, at 6, 12 and 24h and every 24h thereafter	[PC] (μM): D1(481) versus D5(1046 μM): p<0.001Maximum PC incorporation: via PEMT (endogenous) 0.056% versus via CDP-choline (exogenous) 1.26%

RCT: randomized clinical trial;

RDL: days of life;

EN: enteral nutrition;

MP: monitoring period;

GA: gestational age;

OIT: orotracheal intubation;

IG: intervention group;

CG: control group;

[choline]: choline concentration;

PN: parenteral nutrition;

BM: breast milk;

NMR: nuclear magnetic resonance;

HPLC: high performance liquid chromatography;

MS: mass spectrometry;

GC: gas chromatography;

[PC]: phosphatidylcholine concentration;

CDP choline: cytidine diphosphate-choline;

PEMT: phosphatidylethanolamine-N-methiltransferase;

IF: infant formula;

UC: umbilical cord;

NB: newborn;

FNB: Full term newborn;

PTNB: preterm newborn;

SGG: slowest growing group;

FGG: fastest growing group.

## DISCUSSION

 The eight studies selected for review exhibited heterogeneous methodological characteristics, including differences in the material collected, the measuring instruments utilized, and the methodology employed to estimate body or breast milk choline levels in premature newborns. The authors of these studies discussed the importance, dose, and form of choline supplementation in this group, but in different and specific ways. 

 Despite this, the results and discussions converge to similar conclusions, suggesting that current nutritional practices (parenteral and enteral nutrition) are inadequate to fulfil the choline needs of infants born prematurely, particularly those with a lower gestational age at birth. Although the primary source of choline for the human body is exogenous, the dietary supply of choline appears to be a crucial factor in ensuring the optimal homeostasis of these premature infants, particularly given their metabolic immaturity.^11^

 From a physiological point of view, exogenous choline undergoes phosphorylation to phosphocholine, and subsequently converted into CDP-choline, and into phosphatidylcholine via the predominant CDP-choline pathway for phosphatidylcholine biosynthesis. An alternative pathway involves the sequential methylation of phosphatidylethanolamine using S-adenosylmethionine as the methyl donor, resulting in the formation of phosphatidylmonomethylethanolamine, phosphatidyldimethylethanolamine and phosphatidylcholine. This process is catalyzed by the enzyme phosphatidylethanolamine-N-methyltransferase (PEMT).^16^ This is the only means of endogenous choline production and is responsible for approximately 30% of total phosphatidylcholine synthesis in the liver in adults.^8,12^

 However, in newborns under 28 weeks of gestational age, PEMT activity is reduced, and plasma choline concentration decreases to 50% of the umbilical cord concentration within 48 hours of birth, suggesting that these premature infants are dependent on dietary sources and early choline supplementation.^12^

**Figure 2:**
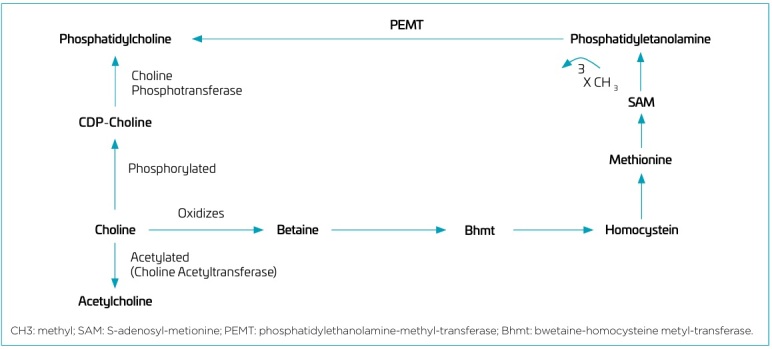
Routes of action and metabolism of choline.

 Choline is a precursor of different metabolites, including the neurotransmitter acetylcholine (ACh), the membrane phospholipids phosphatidylcholine (PC) and sphingomyelin, and the alternative methyl donor betaine for homocysteine-methionine methylation.^17^ Thus, exogenous choline is absorbed and can be phosphorylated into phosphatidylcholine, acetylated into acetylcholine catalyzed by the activity of choline acetyltransferase, and oxidized into betaine (Figure 2).^17,18^

 Phosphatidylcholine is responsible for the structure and function of cell membranes, as well as the formation of bile, surfactant, and lipoproteins.^3,19^ Additionally, phospholipids are essential for intercellular signaling and the hepatic export of very low-density lipoproteins (VLDL).^3,19^ Sphingomyelin is a crucial component of cell membranes, contributing to their structural integrity and signaling capacity.^8^ Acetylcholine is a neurotransmitter involved in memory storage, neural tube development, synapse formation and brain control, as well as muscle control.^3,19,20^ Betaine cannot be reduced to choline and this oxidation pathway acts by reducing the availability of choline to the tissues.^3,19,20^

**Figure 3:**
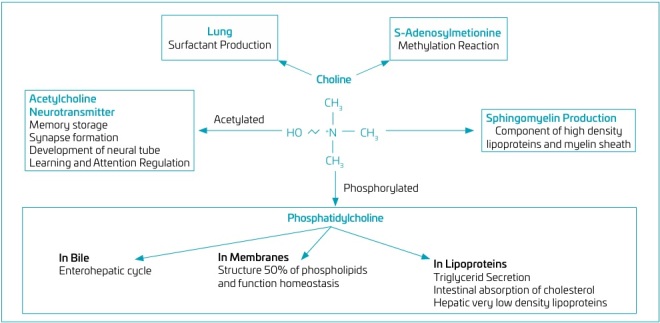
Main functions of choline and its metabolites.

 The essential amino acid methionine is important for the synthesis of S-adenosylmethionine (SAM), which is essential for methylation reactions in different cell compartments. Specifically, the methylation of histones, DNA, and RNA is crucial for epigenetic processes and the regulation of protein synthesis. This underscores the significance of methionine in parenteral nutrition in mitigating the adverse long-term consequences associated with the absence of choline supplementation during the early preterm period.^ 3,8,20 ,21^ The principal functions of choline and its metabolites are illustrated in Figure 3 . 

 Thus, choline participates in critical brain processes during neurodevelopment, including neurotransmitter synthesis (acetylcholine), myelin synthesis (phosphotidylcholine) and epigenetic modification of chromatin (as a methyl donor in homocysteine remethylation pathway).^22^ Despite the ongoing debate in human studies, findings from animal models indicate that dietary choline intake during early life may potentially mitigate the severity of memory impairments in the elderly.^23,24^

 It is reasonable to assume that the nutritional status of the expectant mother has a key influence on the proper development of the fetus. Although all nutrients are important for the developing baby, recent research indicates the importance of adequate choline intake during the periconceptional period, pregnancy and lactation.^25^

 Choline is involved in the formation of a child’s nervous system, supports cognitive development, and reduces the risk of neural tube defects. Current data indicate that most women of reproductive age do not meet the recommended daily intake of choline.^25^ In addition, commercially available prenatal supplements are often not fortified with choline or contain relatively low doses of choline.^26 ,27^

 In light of the growing scientific data confirming the association between choline and fetal development, many researchers emphasize the need to update the official recommendations of obstetric societies. Consideration should be given to the necessity of enteral supplementation of this component during the periconceptional period, throughout pregnancy, and during lactation; campaigns targeting women should also be implemented immediately.^28,29^

 In 1998, the Institute of Medicine (IOM) Food and Nutrition Board published dietary intake recommendations for enteral choline for healthy populations. Due to a lack of evidence, it was not possible to calculate an estimated average requirement for choline; instead, intake recommendations were defined as adequate intakes (AI). The AI for healthy full-term infants aged 0–6 months was set at 125 mg/day, based on the total choline content of 160 mg/L in breast milk and the average intake of 0.78 L/day by newborns and infants in this age group.^8^ However, the amount of choline intake recommended for premature newborns has not yet been established, and there is considerable variability in the scientific literature with respect to this recommendation.^18^

 The typical diets that humans consume provide enough choline. When healthy individuals were provided with a diet deficient in choline for a period of three weeks, they exhibited biochemical alterations consistent with choline deficiency.^16,23^ These alterations included decreased plasma choline concentrations and increased serum alanine transaminase (ALT) activity, which serves as a measure of hepatocyte damage.^30^ Therefore, the minimum values for adults are based on the amount of choline that ensures hepatic metabolism, preventing high concentrations of alanine aminotransferase in the serum, indicating organ function loss.^16^ The AI for children and adolescents is calculated based on the adult AI and the specific growth factor for each age group: AI=adult AI X (child weight/adult weight) 0.75 X (1 + growth factor).^16^

 According to the data available in the literature, the estimated AI values of choline for premature infants using this formula are generally not achieved, as demonstrated by Bernhard et al., who estimated that only 1 to 2% of premature infants using enteral diet associated with parenteral nutrition received this recommendation, especially those with extreme low birth weight.^1^ The absence of choline in the composition of parenteral nutrition, due to the unavailability of this nutrient in the bags supplied by manufacturers, may contribute to this discrepancy. 

 In 2009, the American Society for Parenteral and Enteral Nutrition (ASPEN) published a document recommending the inclusion of choline in parenteral nutrition to prevent liver damage induced by this route of feeding.^24^ One of the functional consequences of dietary choline deficiency in humans has been the development of hepatic steatosis, as the lack of phosphatidylcholine limits the export of excess triglycerides from the liver into lipoproteins.^20,31^ Studies in adults have demonstrated that prolonged parenteral nutrition without choline supplementation is associated with liver morphological abnormalities and altered aminotransferases.^31^ Administration of choline to these patients resulted in improved plasma levels and a reduction in liver fat content.^18,31^ This finding was corroborated by a clinical study of patients who exhibited low plasma concentrations of free choline following treatment with total parenteral nutrition, which lacked additional choline. The subjects received a daily dose of choline chloride, ranging from 1 to 4 grams, over a period of six weeks. Following the administration of choline, plasma concentration increased to within the normal range. However, upon cessation of supplementation, plasma concentration decreased to baseline values.^24,31^

 In this regard, research findings indicate a reduction in serum choline levels that is directly correlated with the number of days during which parenteral nutrition is administered to premature infants, particularly those who exhibit a gradual transition to enteral nutrition. This indicates that the intake of choline has been insufficient in these infants.^11,13^ However, it should be remembered that preterm infants who remain on parenteral nutrition for a long time and do not receive enteral nutrition may be more severely ill. Therefore, clinical severity, and not just gestational age, should be considered in the analysis of studies. 

 Despite the gaps in knowledge about the necessary amount of parenteral choline for premature infants and the lack of products containing this nutrient, there are already recommendations for premature infants receiving a full enteral diet.^32^ According to the US Life Science Research Office (LSRO),^33^ the recommendation varies from 8.4 to 27.6 mg/kg/day; according to Tsang et al.,^34^ from 14.4 to 28 mg/kg/day; and according to the European Society for Pediatric Gastroenterology, Hepatology and Nutrition (ESPGHAN)^10^ and Koletzko et al.,^18^ from 8 to 55 mg/kg/day.^17^ These values were determined using the same formula for children and adolescents.^16^ The differences between the recommendations may be associated with the clinical stability of the newborns and the gestational age and birth weight ranges for which they are aimed.^35^

 One of the Eberhard-Karls-University studies showed that an average enteral dose of 54 mg/kg/day (milk content combined with 30 mg/kg choline chloride supplementation) increased plasma choline in preterm infants from approximately 18 to 35 μmol/L, which is close to the concentration of choline in the umbilical cord at birth. This was at the upper limit of the highest recommendation for enteral feeding of preterm infants.^9^

 Despite carrying out a systematic and careful search of scientific articles to answer the proposed research question, it is important to note that the main difficulties in more definitively assessing the need for choline supplementation in preterm infants are the paucity of articles that have studied choline in preterm infants and the heterogeneity of the samples selected, which makes it difficult to compare the results found in the existing studies. Furthermore, it must be acknowledged that this systematic review included eight articles, four of which were contributed by the same group of authors. This raises concerns about potential bias, even though these studies were rated as 'good' or 'low risk of bias' in the quality analysis of the methods used. Therefore, the results should be interpreted with caution. 

 However, given its relationship with various essential components of metabolism, there is little doubt that the daily supply of choline from the first day of life is critical to the normal development of preterm infants. Although the results of the studies selected here only indicate, but do not allow us to conclude, the amount of choline that should be provided in the PN of these babies, it is important that neonatologists, pediatric nutritionists, nutritionists and pharmacists are aware of the need to include it in the current formulations of PN products and to provide it enterally as soon as possible with formulas or breast milk. 

 Therefore, early enteral nutrition seems to be a crucial point. The inclusion of choline in the parenteral route would need to be better evaluated on the basis of randomised clinical trials with an adequate sample size and with relevant clinical outcomes. Therefore, the gaps in scientific knowledge highlighted here increase the importance of carrying out other intervention studies, in addition to the one identified here, to define the need and the dose to guarantee the infants’ metabolic functions, which seem to be closely and inversely correlated with gestational age (degree of prematurity). 

 Finally, the interpretation of the results of the few existing studies does not allow for a definitive conclusion, but it suggests that choline supplementation should be started in the first days of life of the preterm infant (parenterally) and continued during the initiation and progression of enteral feeding. As for the dose, there is only the current estimate that a dose of 40 to 50 mg/kg/day of choline via enteral feeding seems to be more appropriate to maintain plasma levels close to the amount of choline present in the umbilical cord of the preterm infant. 

## Data Availability

The database that originated the article is available with the corresponding author.
